# 60-Year-Old Man With Recurrent Hemoptysis, Pulmonary Nodules, and Mediastinal Lymphadenopathy

**DOI:** 10.1016/j.chpulm.2025.100141

**Published:** 2025-01-22

**Authors:** Srikant Kashinath Malegaonkar

**Affiliations:** Department of Pulmonary Medicine, All India Institute of Medical Sciences, Nagpur, India

## Abstract

A 60-year-old man with type 2 diabetes mellitus sought treatment at our clinic with episodes of recurrent streaky hemoptysis (about 2 episodes per year for the last 3 years). These episodes resolved with oral antibiotics and empiric oral tranexamic acid given over a short duration of 2 to 3 days. The patient did not smoke and had no other notable medical history, except that one of his coworkers was receiving treatment for pulmonary TB.

## Physical Examination Findings

The patient was afebrile at presentation, with a pulse rate of 78 beats/min. He was normotensive, had a respiratory rate of 17 breaths/min, and oxygen saturation was 98% while breathing ambient air. Physical examination findings were within normal limits, with no abnormal breath sounds heard on chest auscultation.

## Diagnostic Studies

**Figure 1:**
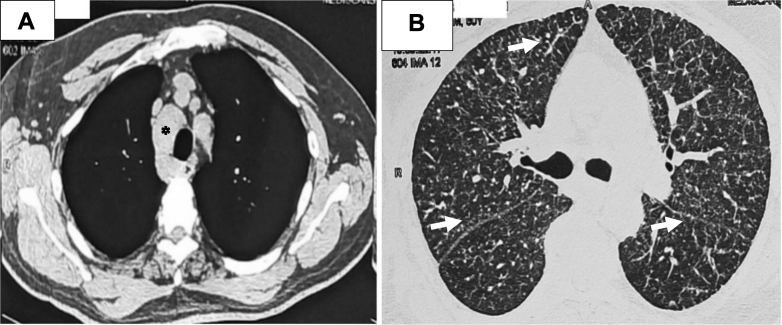
A, Axial section of a chest CT scan with IV contrast showing mediastinal lymphadenopathy (right paratracheal node denoted by black asterisk). B, Parenchymal cuts of an axial section of a chest CT scan showing diffuse perilymphatic nodules (white arrows).

**Figure 2:**
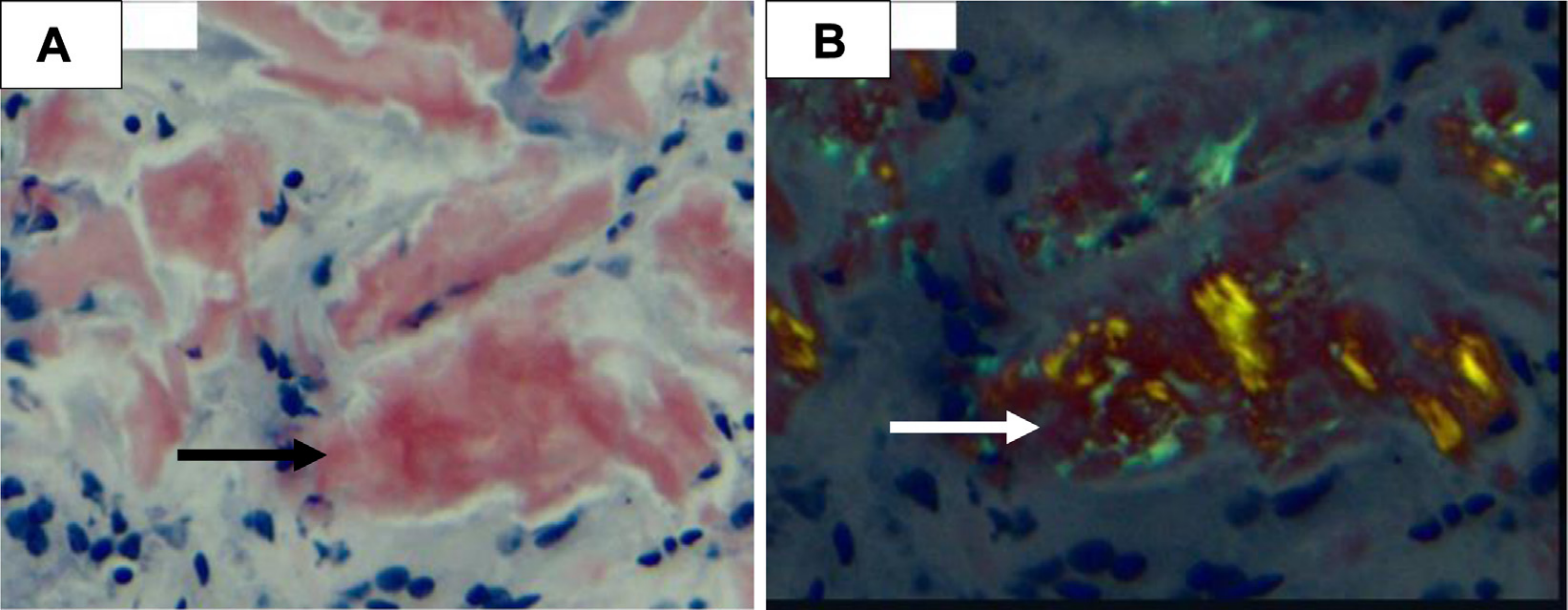
A, Photomicrograph showing a light microscopy section of a lung biopsy specimen with positive Congo red stain material (black arrow). B, Photomicrograph showing a lung biopsy specimen visualized under polarized microscopy showing apple green birefringence (white arrow). Magnification x 400.

A CBC count, biochemistry panel, and coagulation profile were unremarkable. Chest radiograph showed mediastinal widening with bilateral diffuse lung infiltrates. Induced sputum evaluated with Gram stain, acid-fast stain, bacterial culture, and cartridge-based nucleic acid amplification test for *Mycobacterium tuberculosis* complex showed negative results. Contrast-enhanced CT imaging of the chest showed mediastinal lymphadenopathy (involving paratracheal, subcarinal, and bilateral hilar lymph nodes) along with diffuse perilymphatic pulmonary nodules (Fig 1). Serology tests for collagen vascular diseases and systemic vasculitis showed negative results. Endobronchial ultrasound-guided (EBUS) transbronchial needle aspiration (TBNA) from mediastinal lymph nodes was performed along with endobronchial biopsy (EBB) and transbronchial lung biopsies (TBBs) from right lung. The histopathologic examination of the 3 specimens—the EBB, TBB, and tissue cores obtained during EBUS TBNA—revealed the presence of amorphous eosinophilic material extracellularly. This material also exhibited apple green birefringence when stained with Congo red stain (Fig 2). The patient underwent bone marrow aspiration and biopsy, abdominal fat pad biopsy, and serum free light chain assay to determine the extent and cause of underlying disease.

*What is the diagnosis?*

*Diagnosis:* Pulmonary amyloid light chain (AL) amyloidosis secondary to plasma cell dyscrasia

## Discussion

Hemoptysis can occur as a result of diverse causes ranging from infections to malignancy, and CT imaging of the chest, by allowing visualization of tracheobronchial tree and lung parenchyma, is an important investigation for its evaluation. Findings on chest CT imaging along with clues in a patient’s history and physical examination can help the physician to narrow down the differentials for hemoptysis. (1) Perilymphatic nodules on chest CT imaging can occur secondary to benign (sarcoidosis, silicosis) as well as malignant (lymphangitis carcinomatosis) causes. (2) Tissue diagnosis is required to establish an accurate cause, with each of the mentioned differentials for perilymphatic nodules exhibiting their own characteristic histopathologic features on biopsy examination. Among the differentials for perilymphatic nodules, pulmonary amyloidosis can present as tracheobronchial submucosal plaques, pleural effusion, mediastinal lymph node enlargement, diffuse ground-glass opacities, septal thickening, lung cysts, solitary lung nodule, or diffuse lung nodules. Among these, the diffuse alveolar septal form is the most common, and cystic amyloidosis is the least common pulmonary manifestation. (3)

Prior case reports and studies have shown amyloid deposits in mediastinal nodes being detected effectively with EBUS TBNA. (4,5) However, diagnosis of parenchymal and interstitial involvement traditionally is made with video-assisted thoracoscopic surgery or surgical lung biopsy. (6) We were fortunate and this patient’s case is also unique in that all 3 methods used—EBUS TBNA, EBB, and TBB—yielded amyloid deposits favoring multiple pulmonary manifestation without resorting to more invasive tests. EBB was carried out in this patient because pulmonary sarcoidosis was 1 of the differential diagnoses, and combining this procedure with EBUS TBNA plus TBB has been shown to increase diagnostic yield. (7)

After diagnosis based on characteristic histopathologic findings and staining results, further management of pulmonary amyloidosis involves determining fibril protein type, extent of disease, and underlying cause. (8) Amyloidosis involving lungs can be localized (restricted to lungs) or may occur as part of systemic involvement. Plasma cell dyscrasia, chronic inflammatory disorders or chronic infections, and deposition of transthyretin protein either as part of the ageing process or secondary to mutation are causes for pulmonary amyloidosis. (9) Amyloid typing is important because further treatment depends on the type of pathogenic amyloid protein deposited. AL amyloidosis commonly occurs secondary to plasma cell dyscrasia, and thus requires antimyeloma therapies. Other forms of amyloidosis, like serum amyloid protein A amyloidosis, need optimization of underlying inflammatory or infective condition, and forms of hereditary amyloidosis such as amyloid transthyretin amyloidosis require liver transplantation where mutant transthyretin protein is produced. (10) Abdominal fat pad biopsy, bone marrow evaluation, and echocardiography are carried out as part of amyloidosis evaluation to determine systemic involvement. Cardiac involvement in the form of infiltrative or restrictive cardiomyopathy presenting as biventricular wall thickening with normal cavity size and biauricular dilatation can be ruled out with echocardiography.

### Clinical Course

The patient underwent bone marrow aspiration and biopsy, which revealed hypercellular marrow with clonal plasma cells constituting > 1% of cell lines, echocardiography findings were unremarkable, abdominal fat pad biopsy staining showing positive results for amyloid, and serum free light chain assay results were positive. A final diagnosis of AL amyloidosis secondary to plasma cell dyscrasia was made based on these findings. In this patient, empiric treatment with oral tranexamic acid potentially may have impacted the hemoptysis; the evidence for its use in this setting is limited and may have delayed the diagnosis. A hematology department consultation was undertaken to decide on the further course of treatment. A combination of bortezomib, lenalidomide, and dexamethasone was initiated in the patient after discussion of risks and benefits. The patient completed 6 cycles of this regimen with serum free light chain assay results being undetectable along with no further episodes of hemoptysis at follow-up.

## Clinical Pearls

1. *In patients with hemoptysis, chest CT imaging along with clinical evaluation are required to frame the probable differential diagnosis.*
2. *Lung nodules can have diverse causes and require biopsy in most cases to secure correct diagnosis and to initiate appropriate treatment.*
3. *Pulmonary amyloidosis is a rare disease that can have multiple pulmonary manifestations such as septal thickening, lung nodules, lung cyst* *s* *, ground-glass opacity, tracheobronchial submucosal plaques, mediastinal lymphadenopathy, and pleural effusion.*
4. *Traditionally, video-assisted thoracoscopic surgery or surgical lung biopsy have been resorted to for diagnosis of pulmonary amyloidosis. However, less invasive tests such as EBUS TBNA, EBB, and TBB also can help lead to diagnosis.*
5. *Management of pulmonary amyloidosis depends on the cause, the type of fibril protein, and the extent of involvement. Treatment of AL amyloidosis involves chemotherapy and hematopoietic stem cell transplantation (based on eligibility factors).*

## Financial/Nonfinancial Disclosures

None declared.
